# SBOT Turns 90

**DOI:** 10.1055/s-0045-1802355

**Published:** 2025-03-12

**Authors:** Gilberto Luis Camanho

**Affiliations:** 1 Departamento de Ortopedia e Traumatologia, Faculdade de Medicina, Universidade de São Paulo, São Paulo, SP, Brasil

A group of doctors interested in a new specialty from general and pediatric surgery founded the Brazilian Orthopedic Society 90 years ago.

The only thing they had in common was the desire to share knowledge and ignorance about several aspects of the treatment and etiology of diseases and traumas affecting the musculoskeletal system.

Most had some training abroad in different places and had divergent orientations and conducts. They came together to start an inclusive institution, distinct from extractive institutions – terms used by the winners of the 2024 Nobel Prize in Economics to explain why some nations have prospered over the last few years (those with inclusive institutions) and others did not (those with extractive institutions).

The idea was to develop the new specialty, teach it, and disseminate its principles.

With this spirit, the Society developed despite a few disagreements between the founding leaders, always with the idea of teaching by creating new orthopedists in the various national centers.

In 1945, the first medical residency in Brazil was created in orthopedics and traumatology, based on the vision of Godoy Moreira, one of the precursors of the new specialty.

The world was going through one of the most changing times in history, with the emergence of communist doctrine and the reaction of the rest of humanity to it. Great revolutions were beginning in Europe (Germany, Italy, Spain, Russia), parallel to the Industrial Revolution, requiring new doctors capable of rehabilitating traumatized workers and those limited by its post-effects.

The multiplication of orthopedists was proportional to the advance of the specialty, driven by the scourge of World War II, with large-scale traumas and industrial accidents requiring a growing number of specialists. Locomotor system disorders resulting from viruses, such as polio and tuberculosis, created the need for developing specialists in the study of the treatment and rehabilitation of patients with severe limitations as sequelae of these diseases.

This outlined the future of orthopedics: treating and rehabilitating people with musculoskeletal system injuries.

The Brazilian Society of Orthopedics and Traumatology ( *Sociedade Brasileira de Ortopedia e Traumatologia* [SBOT]) has seen significant development in several regions, especially in the Rio de Janeiro-São Paulo region: Pernambuco, Bahia, Goias, Minas Gerais, Parana, and Rio Grande do Sul were the first centers, but soon followed by other states teaching orthopedics and traumatology in their medical schools and sending their young doctors to specialize in centers already structured in the country. The specialty needed a publication to promote it; after unsuccessful attempts, the publication of *Revista Brasileira de Ortopedia* started to occur periodically in Minas Gerais, in 1966. Read and admired by current SBOT members, the journal promotes Brazilian orthopedics worldwide after its indexation in PubMed in 2015. The initial spirit of the founders, i.e., promoting the specialty by teaching it to young doctors, lives on.

In the 1970s, SBOT members, in addition to requiring a minimum of two years of supervised training, decided to create an exam to qualify those who deserved the title of orthopedist, giving rise to the Title of Specialist in Orthopedics and Traumatology ( *Título de Especialista em Ortopedia e Traumatologia* [TEOT]).

This SBOT exam evolved so much that it became an example for several specialties.

In the 1980s, knowledge, as a consequence of all this growth, required a division into areas of study, resulting in the rise of specialties within orthopedics. One by one, the specialties were born and developed like the original Society, always teaching to disseminate their knowledge.

Like branches of a tree that never separate from the trunk, a tree that Nicolas Andry used as a symbol of the specialty correcting pediatric deformities in the book *Orthopedics or the Art of Correcting and Preventing Deformities in Children* , from 1741, the specialties organized themselves.

After 90 years, disagreements, although rarer, persist, as does ignorance about some specialty areas. However, the drive to teach and disseminate information remains growing.

The first CBOT occurred in July 1936 and has been held ever since. This year marks the 57th SBOT meeting. The presidents continue to be elected by SBOT members and still perform their roles of teaching and keeping the unity of the specialty.

Today, SBOT has over 15,000 members and offers numerous modern instruction and teaching systems to them.

It recently opened an international standard training center and laid the cornerstone for a building that will house all its administrative and teaching activities.

I wonder if Rezende Puech, Achilles de Araujo, and Barros Lima would have imagined, in September 1935, that they were founding a society with such a future.

