# Supplementary material for: SBOT Turns 90
Source: Rev Bras Ortop (Sao Paulo). 2025 Mar 12;60(1):s00451802355. doi: 10.1055/s-0045-1802355 (PMC11903114; doi:10.1055/s-0045-1802355)
Supplement: Related texts

# Editorial: SBOT faz 90 anos

Um grupo de médicos que se interessavam por uma especialidade nova, originária da cirurgia geral e da cirurgia pediátrica, fundaram há 90 anos a Sociedade Brasileira de Ortopedia.

A única coisa em comum entre eles era a vontade de compartilhar conhecimentos e a ignorância sobre vários aspectos do tratamento e da etiologia das doenças e dos traumas que afetam o sistema musculoesquelético.

A maioria, com alguma formação no exterior, em locais distintos, tinha orientações e condutas diferentes, e se uniu para iniciar uma instituição de caráter inclusivo, diferente das instituições de caráter extrativo – termos esses utilizados pelos ganhadores do Prêmio Nobel de Economia de 2024, para explicar porque algumas nações prosperaram ao longo dos últimos anos (as com instituições inclusivas) e outras não, (as com instituições extrativas).

A ideia era desenvolver a nova especialidade, ensinando-a e divulgando os seus princípios.

Com esse espírito, a sociedade desenvolveu-se, apesar de alguns desentendimentos entre os líderes fundadores, sempre com a ideia de ensinar formando novos ortopedistas nos diversos centros do país.

Já em 1945, criou-se a primeira residência médica do Brasil, que foi na área de ortopedia e traumatologia, baseada na visão de Godoy Moreira, um dos precursores da nova especialidade.

O mundo passava por um dos momentos de maiores mudanças na história, com o surgimento da doutrina comunista e a reação do resto da humanidade a tal doutrina. Grandes revoluções se iniciavam na Europa (Alemanha, Itália, Espanha, Rússia), paralelas à Revolução Industrial, que exigiam novos médicos capazes de recuperar os funcionários traumatizados e limitados pelas sequelas.

A multiplicação dos ortopedistas era na mesma proporção em que a especialidade avançava, impulsionada pelo flagelo da Segunda Guerra Mundial, os traumas de grande proporção e os acidentes industriais exigiram um aumento no número de especialistas. As doenças do aparelho locomotor provocadas por viroses, como a paralisia infantil e as infecções como a tuberculose, criaram a necessidade de um desenvolvimento dos especialistas no estudo do tratamento e da reabilitação dos pacientes seriamente limitados pelas sequelas dessas doenças.

Assim, desenhava-se o futuro da ortopedia: tratar e reabilitar pessoas com lesões no sistema musculoesquelético.

A Sociedade Brasileira de Ortopedia e Traumatologia (SBOT) teve grande desenvolvimento em várias regiões, destacando-se do eixo Rio-São Paulo: Pernambuco, Bahia, Goiás, Minas Gerais, Paraná, Rio Grande do Sul foram os primeiros centros, mas logo foram seguidos por outros estados que ensinavam em suas escolas médicas, a ortopedia e a traumatologia, e enviavam seus jovens médicos para se especializar em centros já estruturados no país.

A especialidade precisava de um órgão de divulgação, e depois de tentativas infrutíferas a *Revista Brasileira de Ortopedia* , em 1966 nas Minas Gerais, começou a ser publicada com periodicidade. Lida e admirada pelos atuais sócios da SBOT é hoje uma revista que divulga a ortopedia brasileira no mundo, após a sua indexação no PubMed em 2015.

Aquele espírito inicial dos fundadores de divulgar a especialidade ensinando-a aos jovens médicos progredia.

Na década de 1970, os membros da SBOT, além da exigência de um período mínimo de dois anos de treinamento supervisionado, resolveram criar um exame para qualificar quem merecia ter o título de ortopedista e criou-se o Título de Especialista em Ortopedia e Traumatologia (TEOT).

Esse exame da SBOT evoluiu tanto que passou a ser um exemplo para várias especialidades.

Na década de 1980, o conhecimento, como consequência de todo este crescimento, exigiu uma divisão por áreas de estudo e então surgiram as especialidades dentro da ortopedia. Uma a uma, as especialidades foram nascendo e desenvolvendo-se, como a sociedade de origem, sempre ensinando para divulgar.

Como galhos de uma árvore que nunca se dissociaram do tronco, árvore essa que Nicolas Andry usou como símbolo da especialidade que corrigia deformidade em crianças, no livro *Ortopedia ou a arte de corrigir e prevenir deformidades em crianças* , de 1741, as especialidades foram se organizando.

Após 90 anos os desentendimentos, embora mais raros, persistem assim como a ignorância sobre algumas áreas da especialidade, mas o ímpeto de ensinar e divulgar progride intensamente.

O primeiro CBOT foi em julho de 1936 e os demais se sucederam ininterruptamente. Este ano, a SBOT realiza o de número 57, os presidentes continuam a ser eleitos, sempre pela escolha dos sócios, continuam a sua função de ensinar e manter a especialidade unida.

Hoje, a SBOT tem mais de 15.000 sócios e dispõe de vários sistemas modernos de instrução e ensino para os membros.

Recentemente, inaugurou um centro de treinamento de padrão internacional e lançou a pedra fundamental para um edifício que agregará todas as atividades, administrativas e de ensino.

Será que Rezende Puech, Achilles de Araujo e Barros Lima imaginaram, em setembro de 1935, que fundavam uma sociedade com tanto futuro?
